# Serum levels of several molecules that are associated with B cell activation and inflammation are elevated in AIDS-associated non-Hodgkin’s lymphoma (AIDS-NHL) and predict response to treatment

**DOI:** 10.1186/1750-9378-7-S1-O16

**Published:** 2012-04-19

**Authors:** Marta Epeldegui, Daniel P Widney, Anna C Martínez, Deborah Regidor, Larry Magpantay, Ronald Mitsuyasu, Joseph A Sparano, Jeannette Y Lee, Richard F Ambinder, Otoniel Martínez-Maza

**Affiliations:** 1 UCLA AIDS Institute and CARE Center, University of California, Los Angeles, CA, USA; 2 Medicine (Oncology), Albert Einstein College of Medicine, Bronx, NY, USA; 3 Biostatistics, College of Public Health, University of Arkansas, Little Rock, AR, USA; 4 Hematologic Malignancies, Johns Hopkins University, Baltimore, MD, USA

## Background

B cell hyperactivation, as well as loss of immunoregulation of Epstein-Barr virus (EBV) infection, are believed to contribute to AIDS-NHL. Elevated serum levels of several cytokines and immune activation molecules are seen prior to the diagnosis of AIDS-NHL [1]. In this study, we quantified plasma levels of B cell activation-associated molecules (sCD23, sCD27, sCD30, IgE), cytokines (IL-6, IL-10, CXCL13), and *AICDA* expression, prior to and after the initiation of treatment in persons with AIDS-NHL in the AIDS Malignancies Consortium (AMC) 034 study.

## Material and methods

Plasma and PBMC were obtained from AIDS-NHL patients (n=70) in the AMC 034 study, which evaluated treatment of AIDS-NHL with EPOCH chemotherapy and rituximab. Plasma was collected prior to the initiation of therapy, and post-treatment, after the first cycle of chemotherapy, and at 6 and 12 months following completion of treatment. Biomarkers were quantified by ELISA, *AICDA* expression by qPCR.

## Results

Higher pre-treatment plasma levels of most of these B cell activation-associated molecules (IL-6, IL-10, CXCL13, sCD27, sCD30) were seen in AIDS-NHL patients, when compared to HIV+ and HIV-negative reference groups. However, sCD23 levels were lower post-AIDS-NHL than typically seen in the years preceding NHL diagnosis. Additionally, *AICDA* expression in PBMC was not detected in specimens collected after AIDS-NHL diagnosis. Treatment of NHL was seen to result in decreased plasma levels of these molecules, with decreased levels persisting for one year following the completion of treatment. CXCL13 and sCD27 decreased the most after treatment (mean levels went from 487 pg/ml to 310 pg/ml, and 1,080 units/ml to 330 units/ml, respectively) and remained low at one year following initiation of treatment (86.6 pg/ml and 362 units/ml, respectively). Pre-treatment levels of some of these molecules (IL6, IL10, CXCL13) were associated with subsequent response to lymphoma therapy, as were LDH levels and IPI scores. Using a normalizing transformation on CXCL13, CD27 and LDH, logistic regression analysis showed that the only factor significantly correlated with response was CXCL13.

## Conclusions

Biomarkers for AIDS-NHL identified in prior epidemiologic studies were often further elevated post-AIDS-NHL diagnosis, and decreased with treatment for NHL. Importantly, elevated pre-treatment CXCL13 was associated with a poorer subsequent response to treatment, and was a better predictor of response than LDH levels and IPI scores.
